# In Vivo Comparison of Intracameral and Intravitreal Implantation of a Timolol Maleate Miniaturized Injectable Delivery System Device

**DOI:** 10.1016/j.xops.2025.100868

**Published:** 2025-06-26

**Authors:** Chu Jian Ma, Youning Zhang, Daniel A. Bernards, Michele Bloomer, John Dickson, Jayakumar Rajadas, Murty Vyakarnam, Tejal A. Desai, Robert B. Bhisitkul

**Affiliations:** 1 University of California San Francisco, School of Medicine, Department of Ophthalmology, San Francisco, California; 2 University of California San Francisco, Department of Bioengineering and Therapeutic Sciences, San Francisco, California; 3 Oculinea, Inc., San Francisco, California; 4 Stanford University School of Medicine, Stanford, California; 5 Brown University, School of Engineering, Providence, Rhode Island

**Keywords:** Drug delivery, Timolol, Glaucoma, Pharmacokinetics, Microstructured biopolymers

## Abstract

**Purpose:**

Miniaturized injectable delivery system (MIDS) devices engineered for zero-order release of timolol maleate are evaluated for intracameral (IC) or intravitreal (IVT) injection for ocular safety, effects on intraocular pressure (IOP) reduction, and target tissue drug concentrations.

**Design:**

Preclinical study with in vitro testing of MIDS drug delivery devices together with intraocular injection of the devices in normotensive rabbit eyes.

**Subjects:**

Twenty-four New Zealand rabbits received intraocular injections of MIDS devices.

**Methods:**

Timolol maleate MIDS devices were injected IC or IVT into normotensive New Zealand rabbit eyes (n = 24), with weekly ophthalmic examinations and IOP measurements for 8 or 16 weeks. Eight weeks postimplantation, eyes were enucleated for quantification of tissue drug concentrations by liquid chromatography with tandem mass spectrometry and histology on whole globes.

**Main Outcome Measures:**

Drug release pharmacokinetics; ocular safety and biocompatibility; IOP; and blood and target tissue drug concentrations.

**Results:**

At 8 weeks, IOP in experimental eyes was lowered by 11.1 ± 2.9% (n = 5, *P* = 0.019) and 18.1 ± 2.6% (n = 6, *P* < 0.001), for IC and IVT devices, respectively. In extended studies of IVT devices, IOP was numerically lower at 16 weeks by 8.5 ± 5.1% (n = 3, *P* = 0.24). Intracameral versus IVT injections achieved different tissue distributions (in ng/g; except for aqueous in ng/mL): aqueous 28.5 ± 2.7 vs. 4.5 ± 1.2 (*P* < 0.001), vitreous 0.3 ± 0.1 vs. 37.2 ± 11.0 (*P* = 0.010), and ciliary body 14.4 ± 1.8 vs. 50.9 ± 10.8 (*P* = 0.011). Intraocular inflammation and drug- or device-related adverse effects were absent on examinations and histopathology. Blood drug concentration was below the quantitation limit (<0.4 ng/ml).

**Conclusions:**

Intravitreal and IC devices showed similar IOP reductions; IVT injection led to a higher drug concentration in the target ciliary body tissue, and in normotensive rabbit eyes showed general reduction of IOP over 8 weeks, indicating the potential of MIDS technology to address issues of patient adherence with glaucoma eye drops.

**Financial Disclosure(s):**

Proprietary or commercial disclosure may be found in the Footnotes and Disclosures at the end of this article.

Glaucoma is a leading cause of world blindness, with over 70 million people affected, and the number is estimated to increase to almost 112 million by 2040.^1^ Current first-line medication therapies for glaucoma patients rely on the application of daily topical eyedrops, which have the advantages of being patient-administered and noninvasive. But the limitations of eyedrops are well-recognized, foremost being the difficulties that patients have adhering to daily eyedrop regimens that must be continued over many years. Medication adherence is a major barrier to effective glaucoma treatment, with studies showing up to 45% patient nonadherence with eye drop regimens.^2^, ^3^, ^4^ Poor adherence arises from a range of factors: daily application over many years is burdensome, and some patients require multiple eyedrops with complicated dosing regimens; elderly glaucoma patients can have cognitive and mobility issues preventing regular eyedrop use;^5^ patients are often symptom-free, reducing motivation; and self-administering drops can be irritating and manually challenging. Sustained intraocular pressure (IOP) reduction is important for disease control,^6^ and studies have shown that poor compliance leads to worse outcomes—patients with lower scores on medication adherence have been found to have a significantly higher risk of increased visual field deficits.^7^

Topical therapies in glaucoma have other limitations. Many factors can impede penetration of topically applied medication to the target tissues in the eye,^8^ and rapid drug clearance leads to uncontrolled intraocular concentration and therapeutic duration. Topical applications are associated with ocular surface toxicities (such as corneal effects related to eye drop preservatives and excipients) and off-target effects of certain drugs (such as conjunctival injection, eyelid hyperemia, and lash overgrowth). In the case of beta-blocking agents, untoward cardiovascular effects may result from systemic exposure via drainage of excess eyedrop solution through the nasolacrimal system, which can in rare cases lead to serious adverse medical problems.^9^, ^10^, ^11^

The development of ocular drug delivery devices to provide sustained glaucoma therapies offers the potential to overcome these limitations and compliance barriers. Preclinical and clinical programs are underway for several glaucoma sustained-release devices using a range of approaches, including external devices, such as drug-eluting contact lenses and ocular surface devices,^12^, ^13^, ^14^ and intraocular devices.^15^ The bimatoprost intracameral (IC) implant (Durysta, Allergan) is the first intraocular delivery device to be US Food and Drug Administration-approved and available for treatment of glaucoma patients, showing efficacy at 12 weeks in the phase III Bimatoprost Implant in Open-Angle Glaucoma and Ocular Hypertension (ARTEMIS 1) study,^16^ with a majority of patients achieving IOP-lowering through 20 months. With the introduction of sustained glaucoma drug delivery in the clinic, the expansion of this treatment modality holds promise for additional injectable device options with a breadth of drug classes, implantation sites, and drug release profiles and durations.

Here, we describe preclinical studies of a novel technology for the delivery of timolol maleate, an established beta-adrenergic blocker. This miniaturized injectable delivery system (MIDS) device is a biodegradable polycaprolactone-based solid implant; this device is suitable for injection in an office-based procedure and is engineered to attain linear, zero-order drug release kinetics. The MIDS device was evaluated in a normotensive rabbit eye model over 2 to 4 months for safety, IOP-lowering, and ocular tissue drug levels and a comparison of IC and intravitreal (IVT) device implantation was completed to assess the feasibility of these respective approaches.

## Methods

### MIDS Device

**Figure 1:**
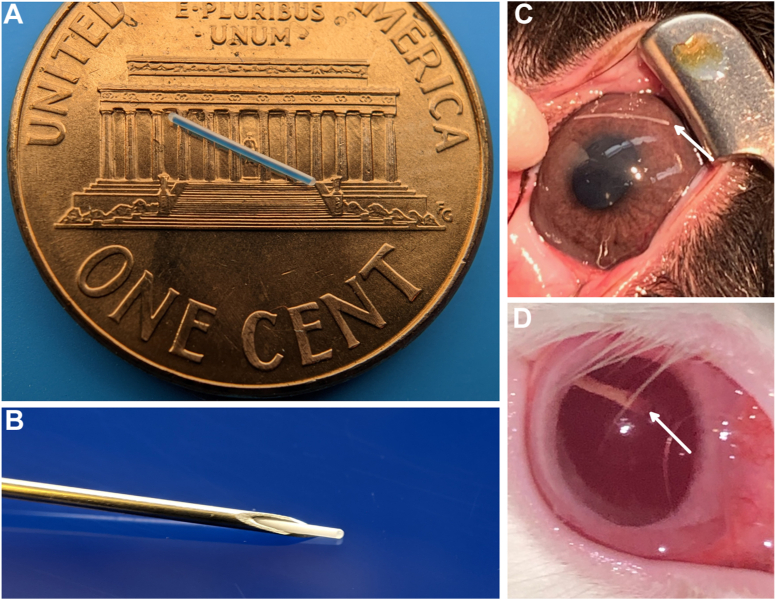
(**A**) Placebo MIDS device. (**B**) Miniaturized injectable delivery system device ejected from thin-walled 22-gauge needle. Photographs of representative rabbit eyes following injection of the timolol MIDS device (arrow) into the anterior chamber (**C**) and vitreous cavity (**D**). MIDS = miniaturized injectable delivery system.

Miniaturized injectable delivery system devices were fabricated as solid cylindrical implants incorporating timolol maleate in a poly(caprolactone) matrix (Fig 1) and were based on methods reported previously.^17^^,^^18^ The fabrication methodology was adapted with a view toward scalability and Good Manufacturing Practice manufacturability based on three unit operations: (1) tubular poly(caprolactone) membranes were dip cast; (2) a solid cylindrical core of timolol maleate and poly(caprolactone) was encapsulated by a tubular membrane; and (3) tubular membrane ends were heat sealed with poly(caprolactone). Nominal MIDS device dimensions were 350 μm in diameter and 6 mm in length. The nominal drug payload was 140 μg, which was suitable for a 6-month zero-order drug-delivery implant. Device diameters were suitable for insertion into 23-gauge thin-walled needles; to facilitate consistent device deployment using standard syringes, devices were loaded into 22-gauge thin-walled needles for injection into the eye for these studies.

### *In Vitro* Release

*In vitro* release was assessed by placing devices (n = 4) in temperature- and pH-controlled fluid chambers of 0.5 ml phosphate buffered saline (PBS; pH = 7.4). Over 28 weeks, the PBS media was fully removed at regular intervals for quantitative drug assays, and the media was replaced with fresh PBS for ongoing studies. To quantify, a standard curve was prepared from a stock solution of timolol maleate (AK Scientific) and serial dilutions. Recovered release media samples were loaded into a 96-well plate and measured with a ultraviolet-visible (UV-vis) plate reader (Molecular Devices SpectraMax M-5 or Tecan Infinite M Nano) at 294 nm. Release rates were calculated by dividing the mass released by the interval since the prior sampling timepoint. Cumulative release was calculated by summing total mass released up to each sampling timepoint.

### Device Implantation

Animal studies were in accordance with the Association for Research in Vision and Ophthalmology (ARVO) Statement for the Use of Animals in Ophthalmic and Vision Research, and the study protocol was reviewed and approved by the University of California, San Francisco Institutional Animal Care and Use Committee. Miniaturized injectable delivery system devices were injected under sterile conditions into the eyes of adult female New Zealand white rabbits under anesthesia (0.03 mg/kg intramuscular buprenorphine and inhalation of 2%–4% isoflurane). All injections were performed by a single investigator. Topical 0.5% proparacaine hydrochloride was applied, followed by ocular wash with 5% povidone iodine solution. Miniaturized injectable delivery system devices were first loaded into a 22-gauge thin-walled needle (McKesson Medical-Surgical) prefilled with balanced salt solution (pH∼7.5). For IC injection, devices were then injected into the anterior chamber through the superior limbus. For IVT injection, a site 2.5 mm posterior to the limbus at 12 o’clock was marked, and the device was injected at the marked site in a direct approach without a scleral tunnel. All device injections were into right eyes; sham injections were performed for control eyes (left eyes) with a 22-gauge needle, and 0.1 cc balanced salt solution was injected without a device. All IOP monitoring studies implanted 1 full-length device. Solely for the purpose of monitoring adverse events, 1 half-length or 2 full-length MIDS devices were also implanted and assessed. For implants of 2 full-length devices, the second device was injected 2 clock hours apart from the first injection site.

### Rabbit Eye Examination with IOP Measurement

Animals were placed under mild sedation with intramuscular buprenorphine 0.03 mg/kg for routine examinations. Anterior segment examination was performed with magnification indirect biomicroscopy using a 20-diopter lens (Volk Inc.) with 0.5% proparacaine eye drops (Sandoz, Novartis Company, NDC 61314-016-01) for anesthesia. Intraocular pressure measurements were performed in an unmasked fashion using an iCare tonometer (Tono-Vet, iCare), with at least three measurements taken for each eye at all time points. Posterior segment examination was done by indirect ophthalmoscopy following pupillary dilation with 2.5% phenylephrine hydrochloride (Bausch and Lomb) and 1% tropicamide (Bausch and Lomb).

### Rabbit Eye Enucleation and Dissection

On the day of enucleation, anterior and posterior segment examinations (with dilation) were performed before euthanasia. Venous blood samples were collected, and then rabbits were anesthetized with buprenorphine and inhalation isoflurane and then sacrificed by intravenous injection of 2 mmol/kg potassium chloride and bilateral thoracotomy. For histology, globes were enucleated and placed in 10% formalin and refrigerated; subsequently, formalin-fixed globes were bisected and processed for paraffin embedding. Ten-micron sections were cut vertically through the pupil optic nerve axis and stained with hematoxylin and eosin.

For tissue drug concentration studies, aqueous fluid was obtained by paracentesis with a 30-gauge needle on a tuberculin syringe, and then globes were dissected to collect cornea, iris, ciliary body, vitreous gel, and retina/choroid. Specimens were placed into Eppendorf tubes and immediately stored at –80°C for subsequent liquid chromatography with tandem mass spectroscopy (LC-MS/MS) analysis. Miniaturized injectable delivery system devices were recovered from the anterior chamber or vitreous cavity using forceps and stored at 4°C for analysis of residual drug content.

### Drug Quantitation via LC-MS/MS

Rabbit fluid samples (blood, aqueous humor, and vitreous humor) were diluted in acetonitrile/methanol and vortexed. The supernatant was then removed and diluted with 0.1% formic acid in water for analysis. For the solid tissue samples (iris, ciliary body, and retina/choroid), the samples were first weighed and homogenized using stainless steel beads. Samples were then processed as above with the fluid samples. For internal standards, timolol-d5 maleate (TRC) was dissolved in dimethyl sulfoxide and diluted in 50% methanol to prepare timolol spiking solutions. For spiked standards, 25 μl of the timolol spiking solution (0.1-100 ng/ml) and 25 μl of the internal standard mix (100 ng/ml for each compound) were added to 25 μl of the control blank ocular tissue or fluid. For test samples, the spiking solution was replaced by 25 μl of 50% methanol to make up the volume.

Samples were analyzed using LC-MS/MS using a Shimadzu LC-20AB HPLC system interfaced with a QTRAP 4000 mass spectrometer (AB SCIEX). Liquid chromatography separations were carried out on an Agilent ZORBAX SB-Phenyl column (50 mm × 4.6 mm, 3.5 μm) using 0.1% formic acid in water and 0.1% formic acid in acetonitrile. The mass spectrometer was operated in positive mode with multiple-reaction monitoring. Data acquisition and analysis were performed using Analyst 1.6.1 software (AB SCIEX).

### Residual Drug Quantification

To determine the presence of residual drug in devices recovered from eyes at sacrifice, MIDS implants were retrieved at dissection and dissolved, timolol maleate was extracted, and the drug content was quantified by UV-vis spectroscopy. First, each device was placed in a 2 ml centrifuge tube, and 40 μl of dichloromethane (Thermo Fisher Scientific) was added. Tubes were agitated until the implant was visibly dissolved. Next, 850 μl of PBS was added to extract timolol maleate from the dichloromethane phase. Phosphate buffered saline was prepared by diluting 10X PBS stock, and pH was adjusted to 7.4 with hydrochloric acid. The tube was vortexed for 2 minutes to ensure full extraction, and the well-mixed solution was then centrifuged at 15 000 rpm for 5 minutes. The aqueous supernatant was extracted for quantification. A standard curve was prepared from a stock solution of timolol maleate (AK Scientific) and serial dilutions. Samples were loaded into a 96-well plate and measured with a UV-vis plate reader (Molecular Devices SpectraMax M-5 or Tecan Infinite M Nano) at 294 nm. If necessary, samples were diluted to ensure they were within the range of the standard curve.

### Data Analysis/Statistical Methods

Normalized IOP was defined as the ratio of experimental IOP (device eye) to sham-control IOP (within-animal fellow eye) and was calculated for each time point and animal individually:

IOP_normalized_ = IOP_MIDS_/IOP_control_

where IOP_MIDS_ is the pressure for the experimental (right) eye and IOP_control_ is the pressure for the control (left) eye at the same time point. This method was included to account for the expected variability of the baseline (control) IOP over time.

Intraocular pressure measurements were done between 9 and 12 am on Thursdays, and the right eye was always measured before the left eye. Standard error of the mean was calculated for *in vitro* release, IOP analyses, tissue drug concentrations, and residual drug analysis. Three MIDS devices were found to have zero residual drug following sacrifice and device retrieval. Based on tissue concentrations and IOP measurements, this result was likely due to faulty construction, and these devices were excluded from all analyses. Intraocular pressure data from animals observed for 16 weeks were used for both 8-week and 16-week endpoints. Student *t* test was used to compare means between different groups. R and Python 3 were used for data analysis.

## Results

### Device Fabrication

**Figure 2:**
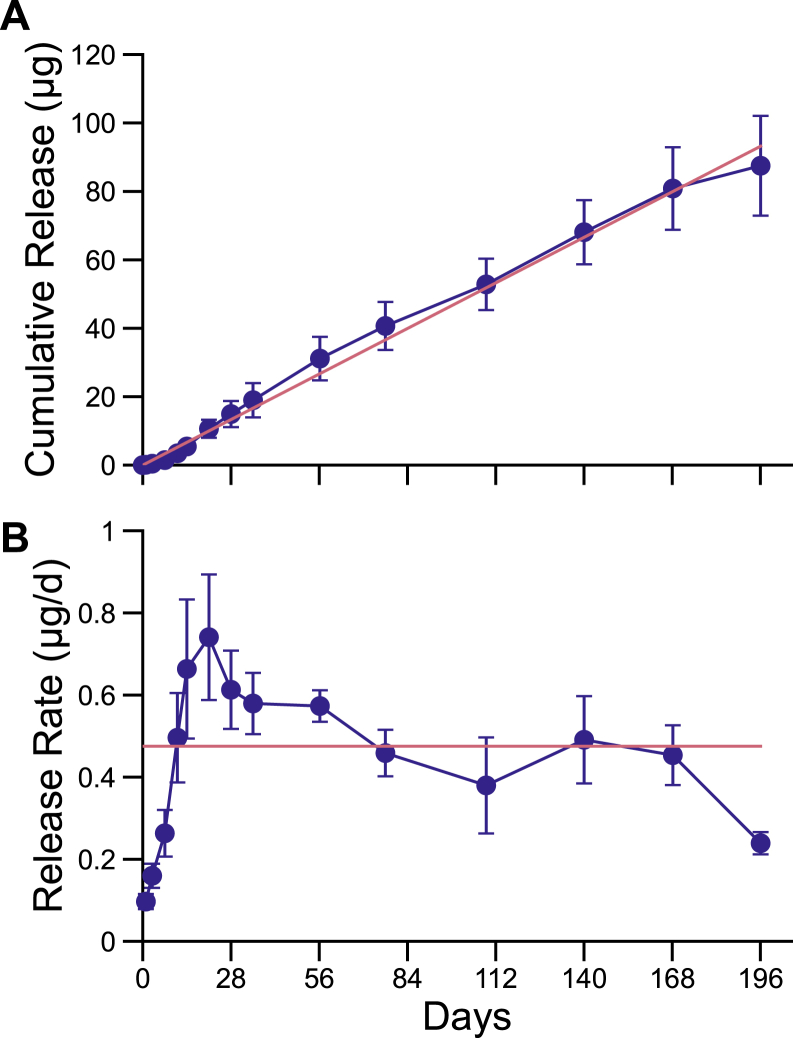
Pharmacokinetics of timolol maleate release from MIDS devices *in vitro* (n = 4). (**A**) Cumulative release curve shows zero-order drug release over 6 months. (**B**) Release rate of MIDS devices shows sustained therapeutic release over 6 months. The best-fit release rate (red lines, *R*^2^ = 0.99) was 0.48 μg/day and was calculated based on the cumulative release. Error bars represent standard error of mean. MIDS = miniaturized injectable delivery system.

Timolol maleate MIDS devices were constructed with a cylindrical form factor, with dimensions suitable for insertion of devices into a thin-walled 23-gauge needle (Fig 1). Devices were tested *in vitro* to determine release behavior (Fig 2). Sustained release was measured over 28 weeks, and a linear fit of the data showed a release rate of 0.48 μg/day (*R*^2^ = 0.99). After *in vitro* testing in controlled fluid chambers, device design was finalized for desired drug release characteristics and subsequent *in vivo* testing in rabbit eyes.

### MIDS Device Safety

**Figure 3:**
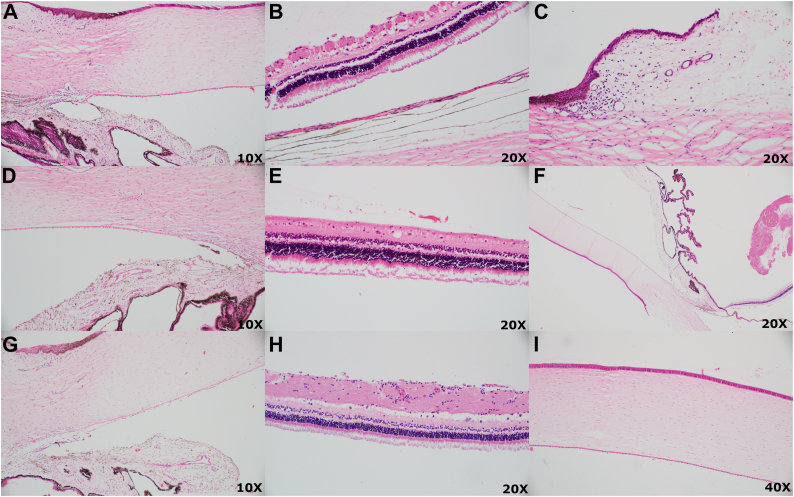
Ocular histopathology studies. (**A**–**C**) Control eye. (**D**–**F**) Experimental eyes with intracameral device implant. (**G**–**I**) Experimental eyes with intravitreal device implant. Hematoxylin and eosin stain was used in all the slides. (**A**, **D**, and **G**): Cornea and angle with normal appearance. (**B**, **E**, and **H**): Retina showing normal architecture. (**C**) Limbus with perilimbal inflammation. (**F**) Vitreous hemorrhage. (**I**) Cornea without edema.

To monitor the safety profile of MIDS devices in rabbits, ophthalmic examinations were performed at baseline, postimplantation day 1, and then weekly to the completion of the studies, with 14 animals to 8 weeks, 6 animals to 10 weeks, and 4 animals to 16 weeks (n = 24 animals). Photographs of the eyes postinjection are shown in Figure 1C and D for IC and IVT devices. Additional animals not included in the IOP study were included for the safety profile. Ocular adverse events included iatrogenic cataract, conjunctival injection, subclinical vitreous hemorrhage, and hyphema (Table 1). There was 1 case of focal cataract that developed at a site with direct mechanical trauma to the posterior capsule and lens during device insertion and 1 focal cataract that developed in a sham injection from the needle hitting the posterior capsule. One case of hyphema was observed following IC injection secondary to iris penetration. One case of vitreous hemorrhage was noted on histology without any abnormal examination findings. Representative histology images are shown in Figure 3 (n = 3 for IC device eyes, n = 3 for IVT device eyes, n = 4 for control eyes). There were no events of hypotony, iritis, vitritis, optic nerve abnormalities, retinal vasculitis, or retinal degeneration detected based on ophthalmic examinations and gross histology. There were no observations of corneal edema in any eyes. Events of conjunctival injection or subconjunctival hemorrhage occurred in most cases following injection for both study and control eyes at day 1 and were attributed to intraocular injection, which resolved by postoperative week 1 to week 2. Devices were retrieved at sacrifice following the study periods, and residual timolol maleate in the devices was measured using UV-vis spectroscopy. All devices at the time of retrieval were intact and displayed no gross or macroscopic degradation, which was expected from the polycaprolactone formulation used in the MIDS devices.

**Table 1:** Adverse Events for 24 Rabbit Test Eyes and 24 Control Eyes

Adverse Event	MIDS Device, N (%)	Sham, N (%)
Cataract (focal)	1 (4.2%)	1 (4.2%)
Conjunctival injection∗	1 (4.2%)	1 (4.2%)
Hyphema	1 (4.2%)	0 (0%)
Vitreous hemorrhage†	1 (4.2%)	0 (0%)
Corneal edema	0 (0%)	0 (0%)
Intraocular inflammation‡	0 (0%)	0 (0%)
Retinal detachment	0 (0%)	0 (0%)
Vascular occlusion	0 (0%)	0 (0%)
Endophthalmitis	0 (0%)	0 (0%)

MIDS = miniaturized injectable delivery system.

∗ At postoperative week 2 examinations or later.

† Detected only on histology.

‡ Iritis or vitritis.

### Intraocular Pressure Effects: IC versus IVT Devices

**Figure 4:**
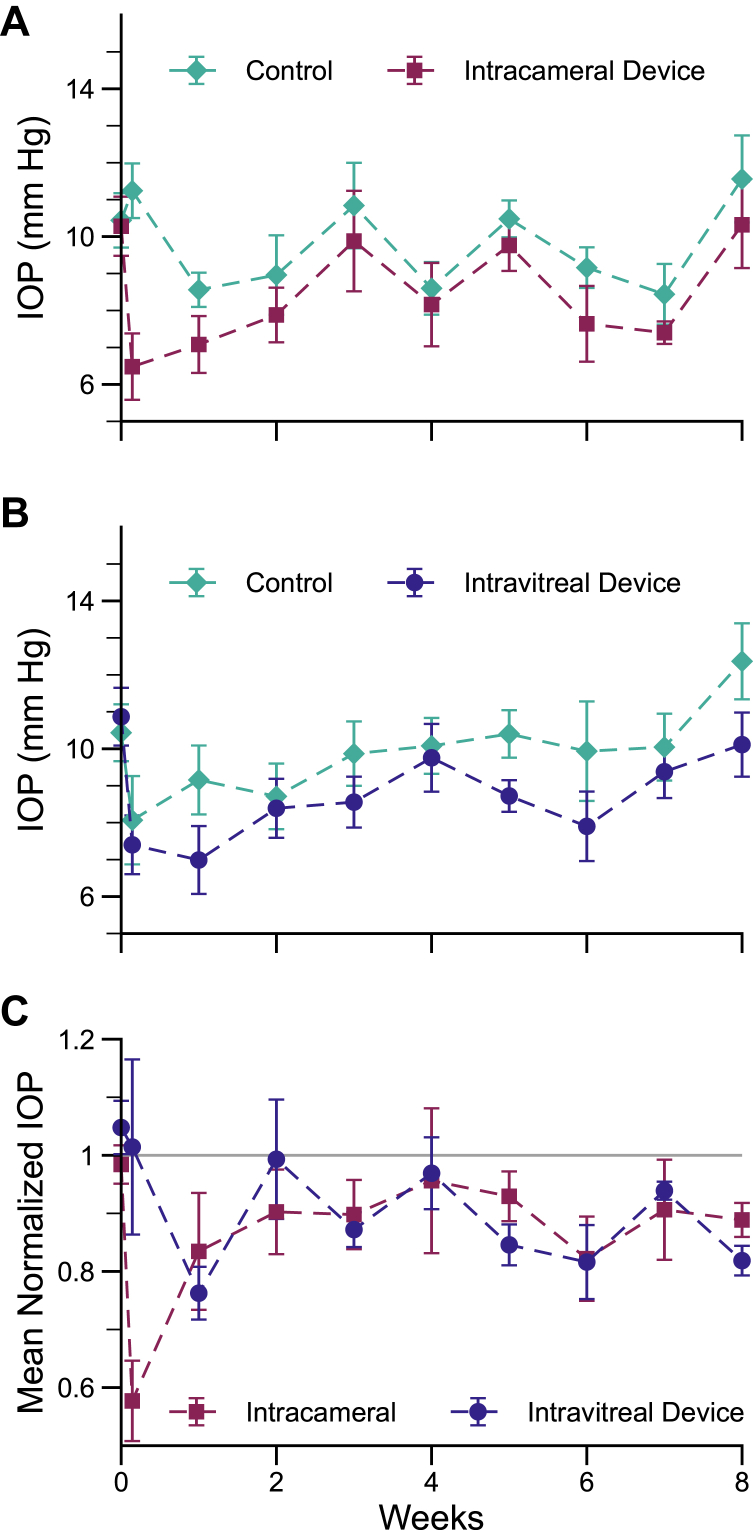
Intraocular pressure for (**A**) IC devices versus sham control and (**B**) IVT devices versus sham control measured over 8 weeks postinjection. (**C**) Normalized IOP for IC and IVT devices. *In vivo* studies demonstrated that at 8 weeks postinjection, IOP in experimental eyes was lowered compared with fellow eye controls by a mean of 11.1 ± 2.9% (n = 5, *P* = 0.019) for IC devices and by a mean of 18.1 ± 2.6% (n = 6, *P* < 0.001) for IVT devices. IOP = intraocular pressure; IC = intracameral; IVT = intravitreal.

Devices were tested at both IC and IVT sites with similar results up to 8 weeks (Fig 4). At 8 weeks postinjection, IOP in experimental eyes was lowered compared with fellow eye controls by a mean of 11.1 ± 2.9% (n = 5, *P* = 0.019) for IC devices and by a mean of 18.1 ± 2.6% (n = 6, *P* < 0.001) for IVT devices. Over the 8-week study period, weekly IOP measurements showed sustained IOP effects following device implantation, with favorable IOP lowering at all but 1 timepoint for IC and for IVT devices. At no timepoint was the study eye IOP significantly higher than the fellow eye control.

### Long-Term IOP Reduction with IVT Devices

**Figure 5:**
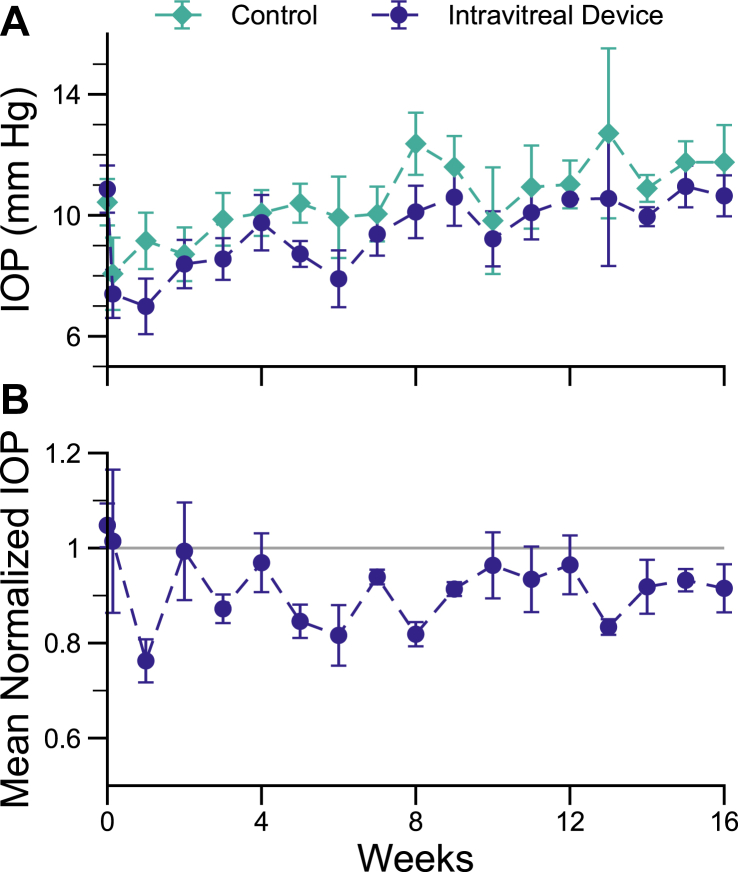
(**A**) Intraocular pressure for IVT devices versus sham control measured over 16 weeks postinjection. (**B**) Normalized IOP for IVT devices. Intravitreal implantation of MIDS devices resulted in a general reduction of IOP over 16 weeks. IOP = intraocular pressure; IVT = intravitreal; MIDS = miniaturized injectable delivery system.

From the 8-week results of IOP reduction for IVT devices, together with therapeutically significant ciliary body drug concentrations, we repeated full-length device IVT implants for longer-term studies through 16 weeks. Results are shown in Figure 5. Intraocular pressure lowering was statistically significant as late as 13 weeks and at 2 out of 8 timepoints from weeks 9 to 16, but not at the final week 16 timepoint (final IOP lowering compared with control of 8.5 ± 5.1%, *P* = 0.24). There was a trend toward sustained IOP reduction, with all but the day 1 timepoint remaining below control over the course of 16 weeks. In addition, all MIDS devices included in this study contained residual timolol payload following extraction from eyes, indicating that drug release is not depleted over 16 weeks.

### Drug Levels at Target Ocular Tissues

**Figure 6:**
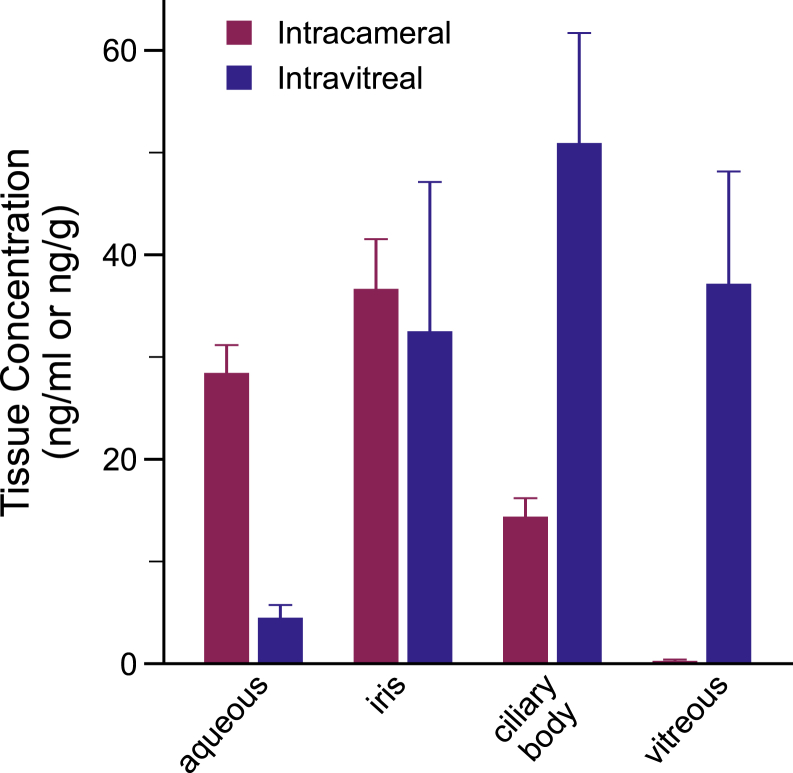
Timolol maleate levels in ocular tissues comparing concentration profiles for IC vs. IVT injections at 8 weeks analyzed by liquid chromatography with tandem mass spectroscopy (in ng/g; except for aqueous in ng/mL): aqueous humor 28.5 ± 2.7 vs. 4.5 ± 1.2 (*P* < 0.001), iris 36.7 ± 4.9 vs. 32.5 ± 14.6 (*P* = 0.77), vitreous 0.3 ± 0.1 vs. 37.2 ± 11.0 (*P* = 0.010), and ciliary body 14.4 ± 1.8 vs. 50.9 ± 10.8 (*P* = 0.011). IOP = intraocular pressure; IC = intracameral; IVT = intravitreal.

Timolol concentrations in various ocular tissues were quantified using LC-MS/MS. At 8 weeks following device implantation, timolol was detected in all ocular compartments. Analysis showed statistically significant differential distribution between IC and IVT devices (Fig 6), with the distribution of drug favoring the ocular tissues in closer proximity to the implant: higher levels of timolol in the aqueous humor for IC implants and higher levels of timolol in the ciliary body and vitreous for IVT implants. Timolol concentrations for IC versus IVT injections were as follows (in ng/mL or ng/g): aqueous 28.5 ± 2.7 vs. 4.5 ± 1.2 (*P* < 0.001), iris 36.7 ± 4.9 vs. 32.5 ± 14.6 (*P* = 0.77), vitreous 0.3 ± 0.1 vs. 37.2 ± 11.0 (*P* = 0.010), and ciliary body 14.4 ± 1.8 vs. 50.9 ± 10.8 (*P* = 0.011). Given that the target tissue for timolol is the ciliary body, further studies focused on IVT implants. Blood samples were collected before sacrifice: drug concentrations in blood were below the quantitation limit (<0.4 ng/ml) at 8 weeks for both groups.

## Discussion

These preclinical studies in rabbit eyes demonstrate the feasibility of MIDS devices for sustained delivery of timolol in the treatment of glaucoma: the small, cylindrical solid implant is injectable with a standard needle into either the anterior chamber or the vitreous cavity; ocular safety outcomes were acceptable with no signs of intraocular inflammation attributable to the drug or device materials; systemic safety was supported with timolol undetected in blood. Intraocular pressure in the normotensive rabbit eye was significantly lowered by 11.1% at 8 weeks with the IC device, and IVT devices showed significant lowering at 8 weeks (18.1%), with a trend for IOP lowering over the course of the 16-week study period. Target tissue drug levels were maintained above estimated therapeutic levels at 8 weeks, with residual device drug present following 16 weeks *in vivo*, indicating capacity for sustained timolol release beyond 4 months.

Miniaturized injectable delivery system devices demonstrated zero-order timolol release *in vitro*, an advance in drug delivery that is distinguished from exponential release kinetics that can arise with devices that mediate drug release via matrix degradation or drug concentration gradient. Linear, zero-order release kinetics can eliminate both initial excess drug peaks and later subtherapeutic tails, but also, by avoiding drug wastage, can maximize the duration of therapy from a necessarily small drug payload. Miniaturized injectable delivery system devices were shown to be injectable into the anterior chamber or vitreous cavity via a 22-gauge thin-walled needle, comparable to in-office procedures performed with established solid implant devices.^19^ With the dimensions of MIDS devices here, it is anticipated to be injectable with a 23-gauge customized injector in the future.

The comparison of IC and IVT MIDS devices demonstrated that the latter route provided 4 times greater tissue levels of timolol drug at 8 weeks at the site of activity, the ciliary body,^20^^,^^21^ which might be expected given that drug clearance in the anterior chamber is greater with turnover of aqueous fluid via trabecular mesh outflow.^22^ Drug distribution of IVT drugs has been modeled for the rabbit eye, demonstrating drug transport from the vitreous to the anterior chamber for clearance through the trabecular meshwork.^23^ Therapeutic levels of timolol maleate in postmortem eye tissues, particularly for the ciliary body, provide confirmatory evidence of therapeutic efficacy. The inhibitor constant for timolol on the cyclic adenosine monophosphate production of the iris-CB complex has been found to be 0.6 nM, or 0.19 ng/mL,^20^ a threshold that was well exceeded at 8 weeks in all study animals for both IC and IVT injections using a single device.^23^ These findings indicate the viability of an IVT timolol device, with the further advantage of minimizing cornea exposure that may contribute to corneal endothelial toxicity and thus limit ongoing treatment with repeated device injections.^24^ Furthermore, IVT injection is a simple, low-risk procedure widely performed by ophthalmologists for retinal therapies, including with established delivery devices such as the dexamethasone implant (Ozurdex, Allergan) and the fluocinolone acetonide implant (Iluvien, Alimera Sciences).

Ocular safety was acceptable in these studies; adverse events observed were primarily related to the device injection procedure itself. No events of significant intraocular inflammation were seen over 16 weeks with MIDS devices from IC or IVT implantation, on either clinical examination or histology. This reinforces the preclinical track record of polycaprolactone in the eye,^25^ and may reflect the relative noninflammatory properties of the polycaprolactone material.^26^^,^^27^ Polycaprolactone materials have a slow degradation profile; bulk degradation is expected to initiate at 9-12 months or more, depending on selected polymer compositions, a timeline that is beyond the scope of the present studies and safety evaluation. Blood levels of timolol were undetectable at 8 weeks with MIDS devices, which is expected to be an advantage of intraocular delivery over topical administration of timolol, which results in systemic absorption through the nasolacrimal system and can have rare but serious side effects on blood pressure and heart rate.^9^, ^10^, ^11^ It may be possible to avoid such side effects via intraocular sustained release therapy, where microgram levels of drug are released in the sequestered space of the eye in direct proximity to target ocular tissues. Another potential benefit is avoiding ocular surface irritation that can be associated with chronic daily eyedrop usage.

While not a disease model for ocular hypertension and glaucoma, the normotensive rabbit eye provides an indication of the efficacy of timolol drug delivery and bioactivity. In this model, control eyes showed notable within-eye fluctuations in IOP over time based on weekly tonometry. Artifactual factors producing variability in IOP monitoring in rabbit eyes have been previously identified, including not only diurnal variation but also factors such as animal handling and water drinking that can introduce wide variability into IOP measurements and are difficult parameters to control.^28^^,^^29^ To offset this variability, IOP was normalized at each timepoint relative to the control fellow eye in each individual animal.

Significant IOP lowering was seen over 8 weeks with both IC and IVT MIDS devices, with lowered IOP at all but 1 weekly timepoint after device implantation, and no observations of higher IOP in test eyes. In longer-term experiments, IVT devices showed a trend for continued IOP lowering; while this was not statistically significant at the final 16-week timepoint, average IOP in study eyes was numerically lower than controls at all timepoints following treatment, and this was statistically significant as late as 13 weeks and at 2 out of 8 timepoints from weeks 9 to 16. While the limitations of the normotensive rabbit eye model have been recognized,^20^ the IOP-lowering effect of timolol in normotensive New Zealand Whites has been observed to be 20% after 30 minutes following topical application at the standard concentration, with a return to baseline at 2 hours.^30^ The magnitude of the IOP-lowering effect observed in these studies was comparable while eliminating the transience of the effect and extending over at least 8 weeks.

In summary, feasibility was demonstrated for this novel MIDS device to provide sustained glaucoma drug therapy for at least 2 months from a single device injection. Miniaturized injectable delivery system devices can be deployed either in the anterior chamber or in the vitreous, offering optionality to curtail effects on the corneal endothelium. In the future, intraocular sustained drug delivery for glaucoma may offer advantages and overcome limitations related to patient adherence with eye drops, thereby improving long-term treatment outcomes for this chronic disease.^30^
